# Prognostic Value of Serum Cystatin C for Predicting Contrast‐Induced Nephropathy in Elderly Individuals Aged Over 80 Years

**DOI:** 10.1155/aiu/9478896

**Published:** 2026-02-27

**Authors:** Xiangxiang Wang, Zhixiang Bian, Rui Zhu, Shunjie Chen

**Affiliations:** ^1^ Department of Nephrology, School of Medicine, Shanghai Fourth People’s Hospital, Tongji University, Shanghai, 200434, China, tongji.edu.cn

**Keywords:** contrast-induced acute kidney injury, cystatin C, percutaneous coronary intervention, prediction

## Abstract

**Objective:**

To explore the predictive value of serum cystatin C (CysC) for contrast‐induced acute kidney injury (CI‐AKI) in elderly individuals.

**Methods:**

The study included 102 elderly patients (80 years) who underwent percutaneous coronary interventions at the Fourth People’s Hospital of Shanghai Affiliated to Tongji University between September 2020 and January 2024. Based on their diagnosis, they were divided into those with CI‐AKI (*n* = 10) and those without CI‐AKI (*n* = 92). A fully automated biochemical analyzer was used to measure the serum CysC levels at baseline and 48 h after coronary angiography or percutaneous coronary intervention in the two groups, and the association of serum CysC levels and other relevant clinical indicators with the occurrence of CI‐AKI was evaluated to determine the predictive value of baseline serum CysC for diagnosing contrast agent‐induced nephropathy based on receiver operating characteristic curves.

**Results:**

Age, serum CysC, and serum creatinine levels were significantly higher in the CI‐AKI group than in the non‐CI‐AKI group. Serum CysC was found to be a predictor of CI‐AKI based on an area under the curve of 0.731 (95% confidence interval = 0.569–0.893).

**Conclusion:**

The occurrence of contrast‐induced nephropathy is related to age, serum CysC, and serum creatinine levels. In particular, serum CysC had high predictive value for CI‐AKI in those aged above 80 years.

## 1. Introduction

Contrast‐induced acute kidney injury (CI‐AKI), which is caused by the use of contrast agents, has become the third most common cause of iatrogenic acquired AKI, only after ischemic kidney injury caused by insufficient renal perfusion or major surgery and kidney injury caused by nephrotoxic drugs. CI‐AKI not only extends the length of hospital stay and increases medical costs but is also correlated with poor prognosis. This condition typically leads to a sharp decline in renal function and an increased risk of death. If not detected or treated, CI‐AKI can result in a rapid deterioration of kidney function, necessitating renal replacement therapy. Additionally, some patients may progress to chronic kidney disease, or even to uremia, requiring lifelong dialysis, which increases the rates of disability and mortality. The increasing use of contrast agents in diagnostic and interventional procedures has led to an increased incidence of CI‐AKI. The elderly, in particular, have a higher risk of CI‐AKI because of the need for more contrast‐enhanced procedures on account of the higher incidence of cardiovascular disease in this population. Early identification of CI‐AKI can guide precise treatment management and, thus, improve patient outcomes.

The current diagnosis of CI‐AKI is based on elevated serum creatinine concentration levels. While serum creatinine is considered a traditional marker of renal function, its levels increase significantly only after approximately 50% of renal function is impaired, rendering it not so sensitive marker for the early detection of CI‐AKI [1]. Moreover, creatinine levels are easily influenced by muscle mass, diet, exercise, and other factors. In elderly patients, increase in age is associated with a decrease in muscle mass, accompanied by a decrease in exercise, as well as associated comorbidities like diabetes and hypertension and the administration of more drugs, all of which are known to affect the production of serum creatinine [2, 3]. Therefore, early biomarkers of acute renal impairment after CAG or PCI are important for the early management and prevention of progression of renal impairment, especially in older patients. Serum cystatin C (CysC) is a novel marker that has been identified and confirmed to have good sensitivity and specificity in evaluating renal function impairment [4]. In particular, Fu Airong et al. found that CysC can be used to predict the risk of CI‐AKI after PCI in elderly patients [5]. However, there are no other studies on CysC as a marker of renal function in the elderly over 80 years old after contrast‐enhanced procedures. In order to fill in this information gap, the present study aims to explore the prognostic value of CysC in CI‐AKI diagnosed in elderly patients over the age of 80 years.

## 2. Materials and Methods

### 2.1. Patient Selection

The study included hospitalized elderly patients over the age of 80 years (age range, 80–98 years; mean ± SD, 85.23 ± 4.005 years) who underwent coronary angiography (CAG) or percutaneous coronary intervention (PCI) at the cardiovascular department of our hospital between September 2020 and January 2024. Their serum creatinine levels fluctuated within the normal range in the 1 month before angiography but did not exceed 20% of the baseline value. All patients were treated with the secondary hypertonic nonionic iobitridol as the contrast agent. The following exclusion criteria were applied for patient selection: acute kidney injury with other clear causes, use of nephrotoxic drugs (such as mannitol) during the perioperative period, end‐stage renal disease, previous kidney replacement therapy, allergy to contrast media, and incomplete clinical data.

This study was approved by the Ethics Committee of Shanghai Fourth People’s Hospital affiliated to Tongji University (Approval no. 2020031‐001), and the patients or their families signed the informed consent form before their inclusion in the study.

### 2.2. General Characteristics and Observation Index

Data on the following variables were obtained: age, sex, baseline creatinine level, baseline serum CysC level, albumin level, hemoglobin level, platelet count, C‐reactive protein level, and underlying diseases (hypertension, diabetes, coronary heart disease, etc.). All the patients received routine hydration treatment before and after surgery.

Fasting venous blood samples were obtained 48 h after CAG or PCI to determine the serum creatinine concentration. According to the 2012 KDIGO guidelines, CI‐AKI was diagnosed if the serum creatinine level increased by 44.2 μmol/L or 25% compared to the baseline within 48 h after the use of contrast agents, assuming that other factors that cause AKI were excluded. Patients were diagnosed and divided into the CI‐AKI group (*n* = 10) and non‐CI‐AKI group (*n* = 92). Relevant indicators were compared between the two groups and analyzed.

### 2.3. Statistical Analysis

Statistical analysis was performed using the SPSS26.0 software. Categorical variables are described as percentages, and comparison between groups was performed with the chi‐square test. Measurement data are described as mean ± SD, and the *t*‐test was used for the analysis of differences between groups. *p* < 0.05 was considered to indicate statistical significance. Receiver operating characteristic (ROC) curve analysis was used to analyze the predictive value of serum creatinine and serum CysC for patients with CI‐AKI.

## 3. Results

### 3.1. Clinical Features

Out of 102 elderly patients (≥ 80 years) who were included, 10 patients (9.80%) developed CI‐AKI, including 5 males and 5 females, and 92 patients (90.2%) did not (non‐CI‐AKI group), including 39 males and 53 females. Their clinical data were compared, and the results are shown in Table 1. There were no statistically significant differences in gender, contrast dose, diabetes, hypertension, or coronary heart disease between the two groups, while there were significant differences in age, as shown in Table 1.

**TABLE 1:** Comparison of clinical data between the two groups.

Parameter	CI‐AKI group (*n* = 10)	Non‐CI‐AKI group (*n* = 92)	*p* value
Age, y	87.50 ± 3.504	84.39 ± 3.367	0.007
Male patients, *n* (%)	5 (50.00%)	39 (42.4%)	0.645
Contrast agent dose (mL)	90.00 ± 30.551	87.07 ± 36.087	0.805
Diabetes cases, *n* (%)	4 (40.00%)	38 (41.3%)	0.937
Hypertension cases, *n* (%)	6 (60.0%)	65 (70.7%)	0.487
Coronary heart disease cases, *n* (%)	10 (100.0%)	87 (94.6%)	0.450

### 3.2. Comparison of Clinical Biochemical Indexes and Prognosis

There were no significant differences in C‐reactive protein, platelet count, hemoglobin, albumin, or other factors between the two groups. However, significant differences were observed in baseline serum creatinine and baseline serum CysC, as shown in Table 2. There was also no significant difference in mortality between the two groups. After angiography, levels of the serum creatinine of 7 patients recovered to preangiography levels within 3 months. However, chronic kidney disease occurred in 2 patients, one of whom did not need renal replacement therapy and one of whom was placed on maintenance hemodialysis. One patient died due to multiple organ failure.

**TABLE 2:** Comparison of test indexes and prognosis between the two groups.

Parameter	CI‐AKI group (*n* = 10)	Non‐CI‐AKI group (*n* = 92)	*p* value
Baseline serum creatinine (μmol/L)	120.600 ± 64.0005	91.246 ± 32.9688	0.019
Baseline serum Cys C (mg/L)	1.7800 ± 0.538	1.3632 ± 0.43602	0.006
C‐reactive protein (mg/L)	13.3900 ± 16.99155	12.4228 ± 20.12142	0.884
Platelet (× 10^9/L^)	203.70 ± 67.385	209.53 ± 77.935	0.821
Hemoglobin (g/L)	115.40 ± 23.505	126.13 ± 18.166	0.088
Albumin (g/L)	36.0490 ± 2.93688	38.7414 ± 6.13165	0.175
Mortality rate, *n* (%)	1 (10%)	7 (7.6%)	0.789

### 3.3. Prognostic Value of CysC for CI‐AKI

**FIGURE 1:**
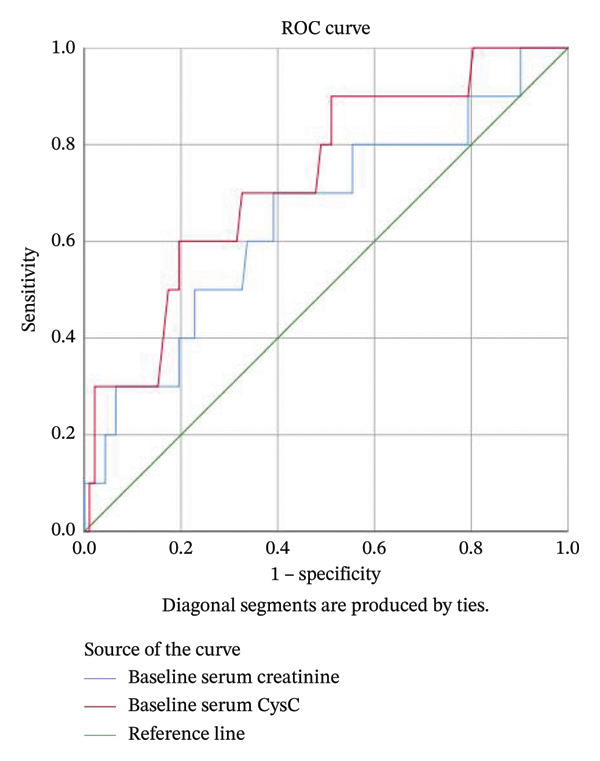
ROC curve of serum CysC and serum creatinine as predictors of CI‐AKI after CAG or PCI.

ROC curve was used to analyze the predictive value of serum creatinine and serum CysC for patients with CI‐AKI, as is shown in Figure 1. The area under the curve (AUC) for serum CysC was 0.731 (95% CI = 0.569–0.893), and it was 0.649 (95% CI = 0.458–0.841) for serum creatinine, as shown in Table 3. Serum CysC had good predictive value for the occurrence of CI‐AKI (*p* < 0.05).

**TABLE 3:** Predictive value of serum CysC and serum creatinine for CI‐AKI after CAG or PCI.

Parameter	Area under the curve	95% CI	*p* value
Baseline serum creatinine	0.649	0.458–0.841	0.122
Baseline serum Cys C	0.731	0.569–0.893	0.017

## 4. Discussion

The present retrospective study of 102 elderly patients over 80 years of age who underwent CAG or PCI showed that the incidence of CI‐AKI in this group was 9.80%, with its occurrence found to be related to age, serum CysC levels, and serum creatinine levels. In particular, serum CysC showed high predictive value based on an ACU value of 0.731.

The incidence of 9.80% observed in this study is higher than the incidence of 7% reported by Alrowaie Fadel et al. in a population of elderly patients over the age of 65 years [6]. This implies that the early identification of high‐risk patients over 80 years old with CI‐AKI is extremely important. As discussed in the introduction, serum creatinine has several limitations with regard to the early detection of CI‐AKI, especially in the elderly. Several biomarkers have been studied as alternatives for the early diagnosis of CI‐AKI, including serum CysC. CysC is an endogenous nonglycosylated protein that is produced at a relatively constant rate and released into the plasma by all nucleated cells in the body. Its molecular weight is very low, and it is easily filtered in the glomeruli. Moreover, it can be completely reabsorbed and catalyzed, without being secreted by the proximal renal tubules. Because of these properties, serum CysC is considered superior to serum creatinine as a marker of AKI. Guoqiang Gu et al. [7] found that CysC before PCI showed a significant difference between patients who later developed contrast‐induced nephropathy and those who did not. Further research has suggested that CysC before PCI could be used as a biomarker of renal function after PCI and that high CysC levels before surgery could predict CIN earlier than serum creatinine levels. For example, Zheng‐Yu Wang et al. investigated the effectiveness of serum CysC in predicting CI‐AKI after intra‐arterial interventional therapy and found that the preoperative serum CysC level has good discriminatory ability for evaluating the risk of CIN after intravascular surgery (AUC = 0.634; 95% CI = 0.526–0.743) [8]. Further, Cecchi et al. [9], in their study on CysC and kidney injury in patients undergoing percutaneous invasive coronary surgery, found that CysC levels were correlated with the occurrence of CIN earlier than changes in creatinine and eGFR. With regard to the elderly population, Fu Airong et al. found that CysC can be used to predict the risk of CI‐AKI after PCI in elderly patients aged 60 years based on an AUC value of 0.81 and optimal cutoff value of 1.5 mg/L, with a sensitivity and specificity of 79.84% and 72.66%, respectively [5]. Similarly, our results also show that serum CysC has high predictive value for CI‐AKI in the elderly population aged above 80 years (AUC = 0.731, 95% CI = 0.5699–0.893). As these are the few studies to demonstrate the predictive value of CysC in this population, the clinical application of CysC as a predictor of contrast‐induced nephropathy in the elderly needs to be investigated further in the future in order to corroborate these findings.

Previous studies have shown that age is a significant risk factor for CI‐AKI [10, 11], as also found in the present study. The higher incidence of CI‐AKI in elderly patients may be related to age‐related renal function decline. That is, with age, the resistance of renal arterioles increases, blood vessels harden, and renal blood flow decreases, and all these mechanisms lead to a decline in renal function. This is compounded by the reduced ability of cells to repair blood vessels with age. All of these factors together increase the risk of CI‐AKI in older patients and reduce the likelihood of rapid kidney recovery [12].

Previous studies have shown that diabetes is a common risk factor for CI‐AKI [13]. However, this study did not find that diabetes was a risk factor for CI‐AKI. Similarly, a prospective open cohort study in 2020 that included 1023 patients with coronary heart disease found that 21.2% of CI‐AKI patients had diabetes and 12.9% had both diabetes and obesity, with diabetes not identified as an independent risk factor for CI‐AKI [14]. As elderly patients tend to have other comorbidities, the risk of diabetes could be masked in this population. In addition, our population may have benefited from the hydration regimen prescribed. Despite this, diabetes as a risk factor for CI‐AKI warrants further study. Other risk factors that warrant further investigation are anemia and hypoproteinemia, as studies have shown that both conditions can increase the incidence of CI‐AKI after PCI [15]. No such correlation was observed in our study population, so investigations in a bigger and wider population could help clarify the potential role of these risk factors.

A number of previous studies have shown that the amount of contrast agent is one of the risk factors for CI‐AKI [16, 17], but this was not observed in the present study. Our finding may be related to the low dose of contrast agent used in all the patients. In fact, Aashaq Hussain Khandy et al. [18] found in their study that the incidence of CI‐AKI increased with increase in contrast medium volume when it reached more than 200 mL. Perhaps, this factor needs further attention, especially in terms of how it might affect serum CysC levels.

Some of the limitations of this study need to be mentioned. The main one is the retrospective, single‐center design, which might have resulted in a bias in the results and might also affect their applicability to a wider population. Second, we were unable to obtain data on urination volume and weight changes, which could have helped determine blood dilution. At the same time, the lack of sensitivity/specificity analysis and the absence of a cutoff value for prediction are also limitations of this study. In the future, multicenter, prospective clinical studies could help better to analyze the incidence of CI‐AKI and related risk factors in elderly patients.

## 5. Conclusions

The present findings demonstrate that serum CysC is a significant predictor of the occurrence of CI‐AKI after contrast‐enhanced procedures in elderly patients over 80 years of age. This requires further verification and analysis in future studies.

## Funding

This work was supported by the General Medical Research Project of Hongkou District Health Commission (HongWei2022‐35) and the Discipline Promotion Program of Shanghai Fourth People’s Hospital (SY‐XKZT‐2020‐1017).

## Conflicts of Interest

The authors declare no conflicts of interest.

## Data Availability

Data can be made available by the corresponding author on reasonable request.
