# Correction to “Prognostic Value of Serum Cystatin C for Predicting Contrast‐Induced Nephropathy in Elderly Individuals Aged Over 80 years”

**DOI:** 10.1155/aiu/9824973

**Published:** 2026-06-14

X. Wang, Z. Bian, R. Zhu, and S. Chen, “Prognostic Value of Serum Cystatin C for Predicting Contrast‐Induced Nephropathy in Elderly Individuals Aged Over 80 years,” *Advances in Urology* 2026 (2026): 9478896, https://doi.org/10.1155/aiu/9478896.

In the article titled “Prognostic Value of Serum Cystatin C for Predicting Contrast‐Induced Nephropathy in Elderly Individuals Aged Over 80 Years,” there was error in the Methods section of the Abstract.

The text “The study included 102 elderly patients (80 years)” was incorrect due to a production error. This should have read: “The study included 102 elderly patients (≥ 80 years)”.

We apologize for this error.

